# Nitrate of Silver in Dysentery

**Published:** 1849-05

**Authors:** 


*Nitrate of Silvr in Dysentery.*—The following is the formula used by Dr. Guyton in dysentery:

℞. Nitrate of silver, ½ a grain to 1 grain;
Water, 10 drachms;
Syrup of poppy heads, 10 scruples;
Syrup of gum Arabic, 13 drachms;
A tablespoonful to be administered every hour.

The first effect of the mixture, according to its author, is to calm the pain and render the evacuations less frequent. It is especially in that form of dysentery called *gastric* or *bilious*, that it appears useful. D. W. Y.

